# High-intensity eccentric training ameliorates muscle wasting in colon 26 tumor-bearing mice

**DOI:** 10.1371/journal.pone.0199050

**Published:** 2018-06-12

**Authors:** Daisuke Tatebayashi, Koichi Himori, Ryotaro Yamada, Yuki Ashida, Mitsunori Miyazaki, Takashi Yamada

**Affiliations:** 1 Graduate School of Health Sciences, Sapporo Medical University, Sapporo, Japan; 2 School of Rehabilitation Sciences, Health Sciences University of Hokkaido, Tobetsu, Japan; Cinvestav-IPN, MEXICO

## Abstract

Eccentric (ECC) contractions are used to maintain skeletal muscle mass and strength in healthy subjects and patients. Here we investigated the effects of ECC training induced by electrical stimulation (ES) on muscle wasting in colon 26 (C-26) tumor-bearing mice. Mice were divided into four groups: control (CNT), CNT + ECC, C-26, and C-26 + ECC. Cancer cachexia was induced by a subcutaneous injection of C-26 cells and developed for four weeks. In experiment 1, muscle protein synthesis rate and mammalian target of rapamycin complex (mTORC) 1 signaling were investigated six hours after one bout of ECC-ES (2 s contraction given every 6 s, 20°/s, 4 sets of 5 contractions). In experiment 2, ECC-ES training, a total of 14 sessions, was performed every other day starting one day after C-26 injection. Compared to the CNT mice, the gastrocnemius muscle weight was significantly decreased in the tumor-bearing mice. This change was accompanied by a reduction in protein synthesis rate and a marked increase in the expression levels of genes including regulated in development and DNA damage responses (REDD) 1, forkhead box protein O1 (FoxO1), muscle-specific E3 ubiquitin ligases atrogin-1, and muscle ring finger 1 (MuRF-1) mRNA. ECC-ES increased the protein synthesis rate and the phosphorylation levels of p70S6K (Thr389) and rpS6 (Ser240/244), markers for mTORC1 signaling, and reversed an upregulation of MuRF-1 mRNA in muscles from C-26 mice. Our findings suggest that ECC-ES training reduces skeletal muscle atrophy in C-26 tumor-bearing mice through activation of mTORC1 signaling and the inhibition of ubiquitin-proteasome pathway. Thus, ECC-ES training might be used to effectively ameliorate muscle wasting in patients with cancer cachexia.

## Introduction

Approximately half of cancer patients experience a syndrome known as cancer cachexia characterized by skeletal muscle wasting. The progression of the cachexia correlates with morbidity and mortality in these patients [1]. Unlike starvation, nutritional supplementation alone is unable to reverse the wasting process [2, 3]. Exercise is a potential intervention that can improve muscle mass in patients with cancer cachexia [4, 5]. In healthy individuals, resistance training is generally recommended to maximize the muscle hypertrophy, because the use of heavy load induces recruitment of fast-twitch fibers that have a greater hypertrophy response compared to slow-twitch fibers [6, 7]. Nonetheless, there are challenges in applying therapeutic exercise, especially when cachexia has occurred, and not all patients are able or willing to perform exercise even at low intensities [8].

In contrast to voluntary contraction, where muscle units are recruited based on the size principle, electrical stimulation (ES) induces the activation of all muscle fiber types and thus is of advantage to activate fast-twitch fibers [9]. Additionally, in those who are unable to undertake the voluntary exercise, ES provides an alternative method for enhancing muscle strength in patients with progressive diseases including cancer [10]. However, optimal parameters and the molecular mechanism behind the effect of ES training have yet to be determined under the cachectic conditions.

Eccentric (ECC) contractions increase muscle mass and strength compared to isometric (ISO) or concentric (CONC) contractions [6]. Moreover, high-frequency stimulation (HFS) is commonly recommended for skeletal muscle hypertrophy [11]. In a recent publication, we reported that ES-induced muscle hypertrophy and the activation of mammalian target of rapamycin complex (mTORC) 1 signaling, a primary regulator of muscle protein synthesis, are modulated by mechanical loading during contraction, but not the contraction mode itself, with a greater gain at HFS than low-frequency stimulation (LFS) [12].

Intriguingly, it has been shown that muscle wasting associated with cancer cachexia is not prevented by ISO- or CONC-ES in tumor-bearing animals [13–15]. Phosphorylation of p70S6K, an indicator of mTORC1 activity, was induced by CONC-ES at LFS training in control, but was blocked in cachexic muscles, suggesting an impaired anabolic response to ES under cachexia [16]. In contrast, recent studies demonstrated that ECC-ES attenuates muscle atrophy in tumor-bearing mice [11, 13]. In these studies, however, the torque production was not measured during ECC-ES and hence the role of loading intensity in prevention of cachexia-induced muscle wasting has not been elucidated.

The control of muscle mass depends on the dynamic balance between anabolic and catabolic processes [17, 18]. Glucocorticoid signaling plays a critical role in cancer-induced muscle atrophy [19]. Glucocorticoid has antianabolic and catabolic effects and promotes degradation via the activation of ubiquitin-proteasome and autophagy pathway in fast-twitch fibers with less or no impact on slow-twitch fibers [20, 21]. Among these, the ubiquitin-proteasome pathway is responsible for the degradation of the majority of soluble and myofibrillar proteins [22].

In the present study, we hypothesized that ECC-ES training would prevent muscle wasting in colon-26 tumor-bearing (C-26) mice, and that this intervention would reverse the suppressed anabolic signaling associated with the wasting processes during the progression of cachexia. Our results showed that ECC-ES ameliorates muscle atrophy in C-26 mice. Moreover, this beneficial effect could be attributed to an activation of mTORC1 signaling that may be induced in an intensity-dependent manner even under the cachexia condition.

## Materials and methods

### Ethical approval

All experimental procedures were approved by the Committee on Animal Experiments of Sapporo Medical University (No.15-044_17–078). Animal care was in accordance with institutional guidelines.

### Experimental design

To examine the acute and chronic effects of ECC-ES on muscle wasting in C-26 mice, we performed two separate experiments.

#### Experiment 1

We first assess the acute effects of ECC-ES on protein synthesis rate and anabolic signaling in skeletal muscle from C-26 mice. Male CD2F1 mice (8 week old, n = 13) were supplied by Sankyo Labo Service (Sapporo, Japan) and were given food and water ad libitum and housed in an environmentally controlled room (24 ± 2°C) with a 12-h light-dark cycle. Daily food intake was measured during the experimental periods in all animals. C-26 cells were provided by the RIKEN BRC through the National Bio-Resource Project of the MEXT, Japan. Mice were randomly assigned to control (CNT) (n = 6) and C-26 (n = 7) group. The C-26 cells were cultured *in vitro* with RPMI-1640 supplemented with 10% (vol/vol) fetal bovine serum and 1% penicillin/streptomycin, and incubated at 37°C with 5% CO_2_. Cancer cachexia was induced by subcutaneous injection of 5×10^5^ C-26 cells diluted in 0.1 ml phosphate buffered saline into right flank. Four weeks after C-26 injection, a single bout of ECC-ES was performed on the left leg (CNT + ECC and C-26 + ECC group) using electrical stimulator (Nihon Kohden, Japan). The right legs served as a non-ES control. Six hours after an ECC-ES bout, mice were anaesthetized with 2% isoflurane through face masks connected to a vaporizer and were killed cervical dislocation. Muscle protein synthesis rate and mTORC1 signaling were investigated in gastrocnemius muscle.

### Eccentric contractions

**Fig 1:**
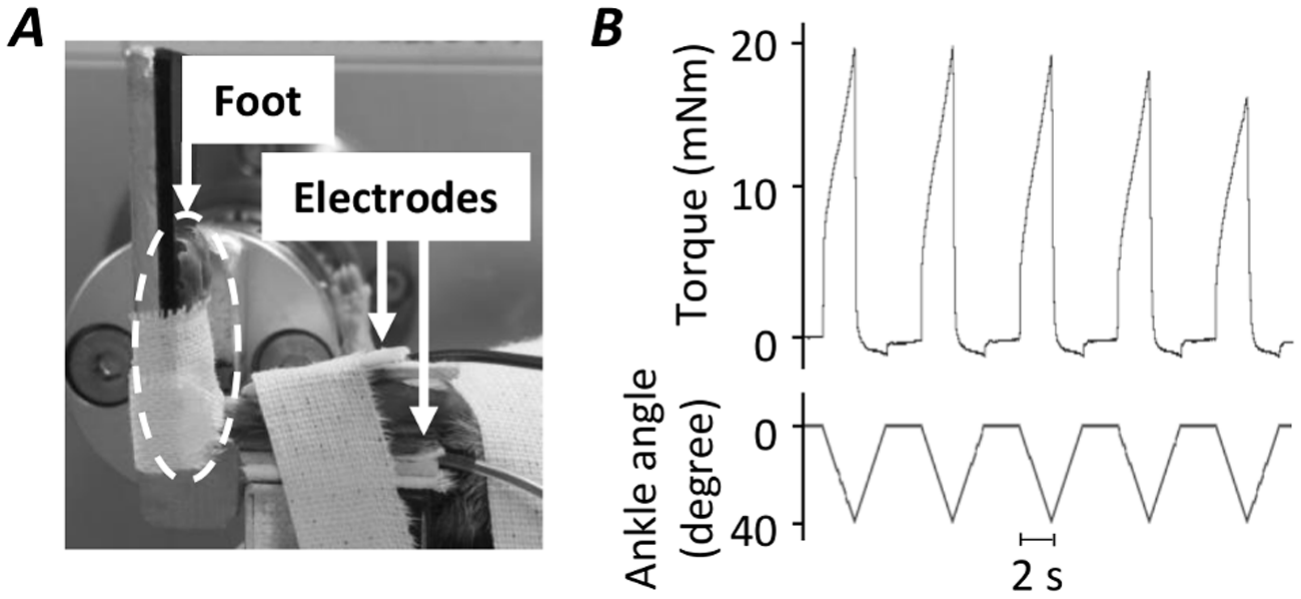
Procedure for ECC training. The foot was placed at 0° of dorsal flexion. Surface electrodes were placed on the front and back surface of lower leg and plantar flexor muscles were stimulated supramaximally (45 V) for 2 s given every 6 s (***A***). ECC contractions comprised forced dorsiflexion from 0° to 40° at 20°/s during electrical stimulation. Typical torque traces of plantar flexion and ankle angle of dorsal flexion during eccentric contractions (***B***).

Under isoflurane anesthesia, mice were placed supine on a platform and their left foot was secured in a foot plate connected to a torque sensor (S-14154, Takei Scientific Instruments, Japan) at an angle of 0° dorsiflexion (i.e., 90° relative to the tibia). ECC contractions comprised forced dorsiflexion from 0° to 40° at 20°/s during ES (Fig 1A). Plantar flexor muscles were stimulated supramaximally (45 V) for 2 s given every 6 s via a pair of surface electrodes. Stimulation parameters were set as follows: 0.5 ms monophasic rectangular pulse, 100 Hz stimulation frequency. Each session consisted of 4 sets of 5 contractions at 5 minutes intervals. Fig 1B shows typical torque and the ankle angle traces during ECC contractions.

### Measurement of protein synthesis

Muscle protein synthesis rate was measured using a nonradioactive technique as described previously [23]. Briefly, puromycin (0.04 μmol/g body weight dissolved in 100 μl of PBS) was intraperitoneally injected into each animal, and then the gastrocnemius muscle was excised and quickly frozen in liquid nitrogen at exactly 30 min following the puromycin injection. The amount of puromycin incorporation into nascent peptide chains was determined by immunoblot analysis (see below).

### Immunoblots

Immunoblots were performed using anti-puromycin (MABE343, Merck Millpore, Damstadt, Germany), anti-total 70 kDa ribosomal S6 kinase (p70S6K), anti-p-p70S6K (Thr389), anti-total ribosomal protein S6 (rpS6), and anti-p-rpS6 (Ser240/244) (Cell Signaling Technology, Beverly, MA) in experiment 1 and anti-LC3B (ab63817, Abcam, Cambridge, MA), anti-beclin-1 (3738, Cell Signaling, Beverly, MA), anti-p62 (ab56416, Abcam, Cambridge, MA), anti-actin (A2172, Sigma Aldrich, Japan), anti-tropomyosin (Tm) (T9283, Sigma Aldrich, Japan), anti-total troponin I (TnI) (4002, Cell Signaling, Beverly, MA), anti-fast type troponin T (fTnT) (T6277, Sigma Aldrich, Japan), anti-desmin (ab32362, Abcam, Cambridge, MA), and anti-GAPDH (010–25521, Wako, Japan) in experiment 2.

Muscle pieces were homogenized in ice-cold homogenizing buffer (40 μl/mg wet wt) consisting of (mM): Tris maleate, 10; NaF, 35; NaVO_4_, 1; 1% Triton X 100 (vol/vol), and 1 tablet of protease inhibitor cocktail (Roche) per 50 ml. Protein content was determined using Bradford assay [24]. Aliquots of the whole muscle homogenates (20 μg) were then diluted with SDS-sample buffer (mM): Tris HCl, 62.5; 2% SDS (wt/vol); 10% glycerol (vol/vol); 5% 2-mercaptoethanol (vol/vol); 0.02% bromophenol blue (wt/vol). Proteins were applied to a 4–15% Criterion Stain Free gel (BioRad). Gels were imaged (BioRad Stain Free imager), and then proteins were transferred onto polyvinylidine fluoride membranes. Membranes were blocked in 3% (wt/vol) non-fat milk, Tris-buffered saline containing 0.05% (vol/vol) Tween 20, followed by incubation with primary antibody, made up in 1% (wt/vol) bovine serum albumin (BSA) overnight at 4 °C. Membranes were then washed and incubated for 1 h at room temperature (24°C) with secondary antibody. Images of membrane were collected following exposure to chemiluminescence substrate (Millipore) using a charge-coupled device camera attached to ChemiDOC MP (Bio-Rad), and Image Lab Software (Bio-Rad) was used for detection as well as densitometry.

#### Experiment 2

To investigate the chronic effects of ECC-ES training on muscle mass and catabolic signaling in skeletal muscle from C-26 mice, male CD2F1 mice (5 week old, n = 10) were randomly assigned into CNT and C-26 groups (n = 5 in each group). ECC-ES training was started one day after C-26 injection and performed every other day for four weeks (i.e., a total of 14 sessions). Mice were killed cervical dislocation under isoflurane anesthesia and the gastrocnemius muscles were dissected from each animal 24 hours after the last ECC-ES session to avoid the effect of changes in fluid content on the muscle wet weight [25].

### Quantitative real-time PCR

Real-time PCR was used to quantify the mRNA levels for regulated in development and DNA damage responses (REDD) 1, forkhead box protein O1 (FoxO1), muscle-specific E3 ubiquitin ligases atrogin-1 and muscle ring finger 1 (MuRF-1) in frozen gastrocnemius muscle tissue. Briefly, total RNA was isolated from muscle samples using RNeasy Fibrous Tissue Mini Kit (Qiagen, Valencia, CA) according to manufacturer’s instructions. Following isolation, the RNA was quantified using UV spectrophotometry (Nanodrop Light, Thermo Fisher Scientific, Waltham, MA). Total RNA was reverse-transcribed to cDNA using Prime Script RT Reagent Kit (Takara, Japan). Synthesized cDNA was then amplified on the Thermal Cycler Dice^®^ (Takara) with Premix Ex Taq^™^ kit (Takara, Japan). The following Taqman Probes (Applied Biosciences^™^, Carlsbad, CA) were used: mouse REDD1 (Ddit4, Mm00512504_g1), mouse FoxO1 (Mm00490672_m1), mouse atrogin-1 (Ebxo32, Mm00499523_m1), mouse MuRF-1 (Trim63, Mm01185221_m1), mouse glyceraldehyde-3-phosphate dehydrogenase (GAPDH) (Mm99999915_g1). All samples were run in duplicate. Relative amounts of target mRNA was determined using the comparative threshold cycle method (ΔΔCT). Expression of target genes was normalized to the corresponding expression level of GAPDH.

### Myofibrillar protein concentration

The myofibrillar protein concentration in gastrocnemius muscles were determined as previously described [26]. Briefly, muscle pieces were homogenized in ice-cold *buffer 1* (20 μl/mg wet wt) consisting of 20 mM imidazole, 0.25 mM phenylmethylsulfonyl fluoride, pH 7.4. Following a 15 min centrifugation at 12,000 *g* at 4°C, the supernatant was removed, and the remaining pellet was homogenized with *buffer 1* (20 μl/mg wet wt), and centrifugation was repeated. The remaining pellet was then homogenized in *buffer 2* (20 μl/mg wet wt) consisting of 0.5% (vol/vol) trifluoroacetic acid, 1 mM tris (2-carboxyethyl phosphine) hydrochloride followed by a 15 min centrifugation at 12,000 *g*. The supernatant was collected, and the remaining pellet was homogenized in *buffer 2* (20 μl/mg wet wt), and centrifugation was repeated. The combined supernatants comprised the fraction enriched in myofibrillar proteins. Protein concentration was determined using Bradford assay [24] with BSA as the standard.

### Statistics

Data are presented as mean±SE. Student’s *t*-test was used to compare the tumor-free body weight in experiment 1 and 2 and the gastrocnemius muscle weight and daily food intake in experiment 1 between CNT and C-26 mice. A two-way ANOVA was used to determine statistically significant differences in gastrocnemius muscle weight in experiment 2 and the expression levels of mRNA and protein in experiment 1 and 2. The Student-Newman-Keuls *post hoc* test was used to isolate the significantly different mean. A *P* value less than 0.05 was regarded as statistically significant. Statistical testing was performed with SigmaPlot (version 13, Systat Software, Inc, CA).

## Results

### Body and muscle weights

Cachexia is defined as a loss of 5% of body weight, including muscle and fat mass, given an underlying disease. Tumor-free body mass in C-26 mice was 17% lower than CNT mice both in experiment 1 and 2 (Table 1). C-26 mice had smaller gastrocnemius muscle mass (-11% and -12% in experiment 1 and 2, respectively) compared with CNT mice. Thus, these data indicate that C-26 tumor bearing induced the cancer cachexia and the magnitude of muscle wasting was similar between the two experiments. Importantly, in experiment 2, ECC-ES training increased the gastrocnemius muscle mass irrespective of cachexia.

**Table 1:** Effects of tumor-bearing and/or eccentric training on body and muscle mass in control and colon 26 mice.

	Experiment 1		Experiment 2	
	CNT (n = 6)	C-26 (n = 7)	CNT (n = 5)	C-26 (n = 5)
Tumor-free body mass (g)	25.9 ± 0.8	21.4 ± 0.3^†^	21.8 ± 0.2	18.2 ± 0.4^†^
Tumor mass (g)		5.2 ± 0.4		5.7 ± 0.7
GAS wet weight (mg)				
untrained	119.7 ± 3.8	106.9 ± 1.8^†^	101.1 ± 2.0	89.3 ± 6.6*^a^
ECC			106.8 ± 1.0^#^	94.8 ± 3.6*^#^^c^

Values are means ± SEM. CNT: control, C-26: colon 26, ECC: eccentric training, GAS: gastrocnemius muscle. Statistical significance was set at *P* < 0.05: main effect of *C-26 and

^#^ECC; difference versus

^†^CNT,

^a^CNT-untrained, and

^c^CNT-ECC.

It has been reported that C-26 mice do not develop anorexia [27]. In line with this, average daily food intake did not differ between CNT and C-26 mice (2.7 ± 0.1 g and 2.6 ± 0.1 g). Thus, muscle atrophy in C-26 mice was not due to anorexia.

### ECC-ES training activates protein synthesis and the mTORC1 signaling in gastrocnemius muscles from C-26 mice

**Fig 2:**
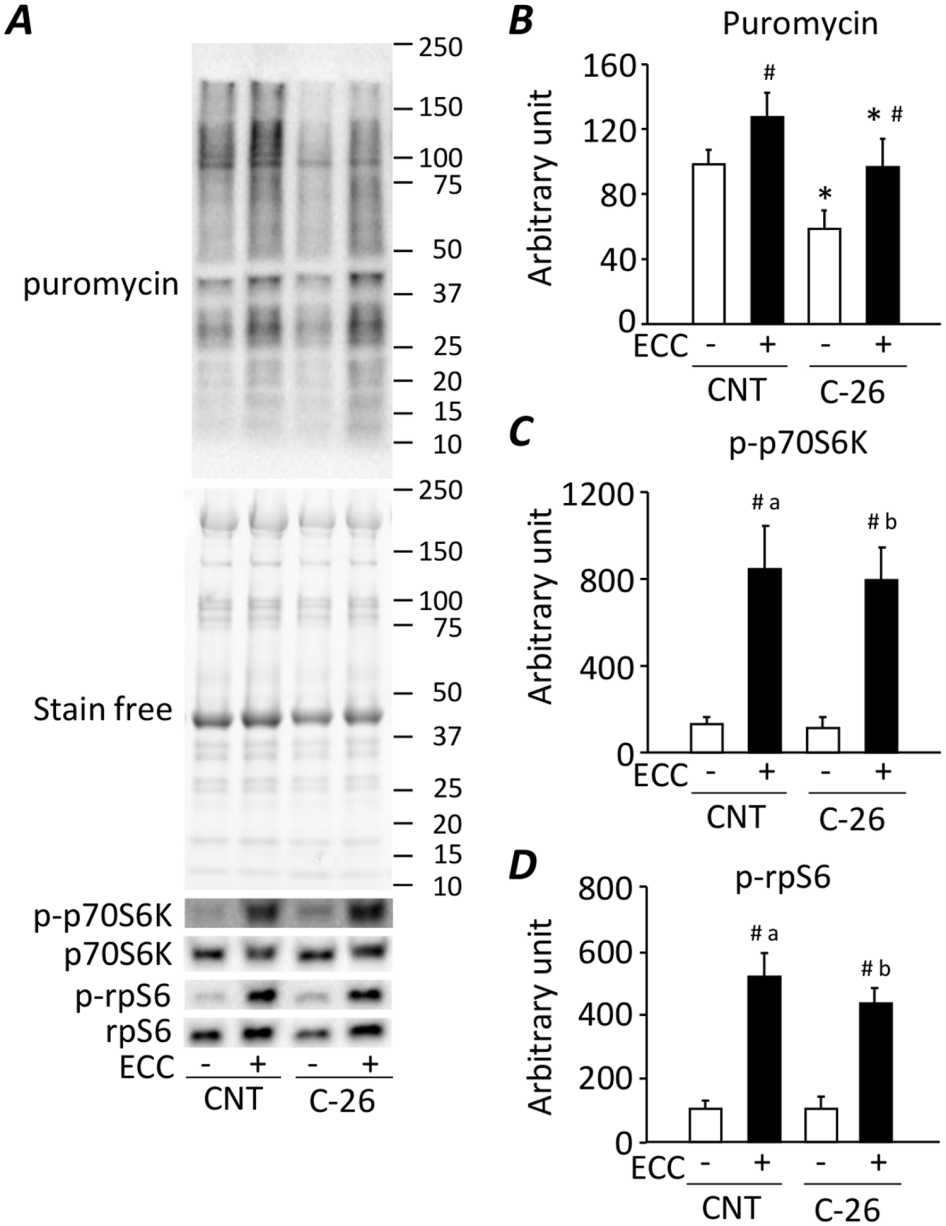
ECC-ES training activates protein synthesis and the mTORC1 signaling in gastrocnemius muscles from C-26 mice. (***A***) Representative western blots for puromycin, total and phosphorylated p70S6K Thr389 (p-p70S6K) and rpS6 Ser240/244 (p-rpS6) in control (CNT) and C-26 mice at 6 hours after one bout of ECC-ES. The expression levels of puromycin was normalized to the whole proteins in stain-free images (***B***). The levels of p-p70S6K (***C***) and p-rpS6 (***D***) were normalized to total p70S6K and rpS6 content, respectively. Data show mean ± SEM for 5–7 muscles per group. Statistical significance was set at *P* < 0.05: main effect of *C-26 and ^#^ECC; difference versus ^a^CNT-untrained and ^b^C-26-untrained.

Protein synthesis rate was reduced in muscles from C-26 mice compared to CNT mice (Fig 2A & 2B). Notably, protein synthesis was induced by a single bout of ECC-ES irrespective of cachexia. Moreover, ECC-ES induced marked increase in the phosphorylation levels of p70S6K Thr389 and rpS6 Ser240/244 in C-26 group (Fig 2A, 2C and 2D). The magnitude of the activation in these mTORC1 signaling molecules was similar in both CNT and C-26 mice.

### ECC training inhibits increases in E3 ubiquitin ligase MuRF1 mRNA in gastrocnemius muscles from C-26 bearing mice

**Fig 3:**
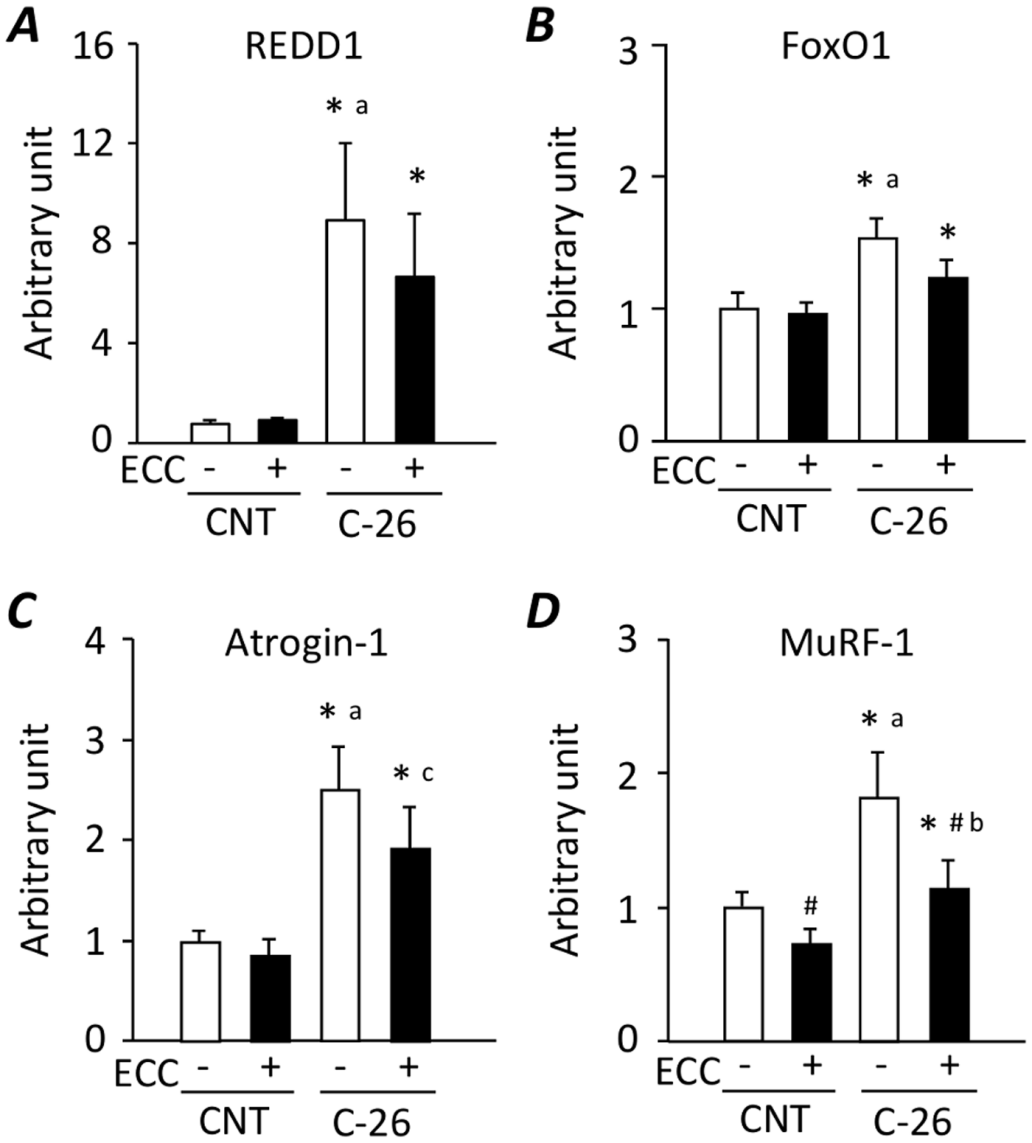
ECC training inhibits increases in E3 ubiquitin ligase MuRF1 mRNA in gastrocnemius muscles from C-26 bearing mice. Expression levels of regulated in development and DNA damage responses (REDD) 1 (***A***), forkhead box O (FoxO) 1 (***B***), atrogin-1 (***C***), and muscle ring finger protein 1 (MuRF-1) (***D***) mRNA in gastrocnemius muscles from control (CNT) and C-26 mice with or without ECC training. Data show mean ± SEM for 5 muscles per group. Statistical significance was set at *P* < 0.05: main effect of *C-26 and ^#^ECC; difference versus ^a^CNT-untrained, ^b^C-26-untrained, and ^c^CNT-ECC.

Expression levels of REDD1, FoxO1, atrogin-1, and MuRF-1 mRNA were significantly increased in gastrocnemius muscles from C-26 mice compared with CNT mice (Fig 3). ECC-ES training inhibited an upregulation of MuRF-1 mRNA expression in muscles from C-26 mice.

### Increase in autophagy-related protein in gastrocnemius muscles from C-26 bearing mice

**Fig 4:**
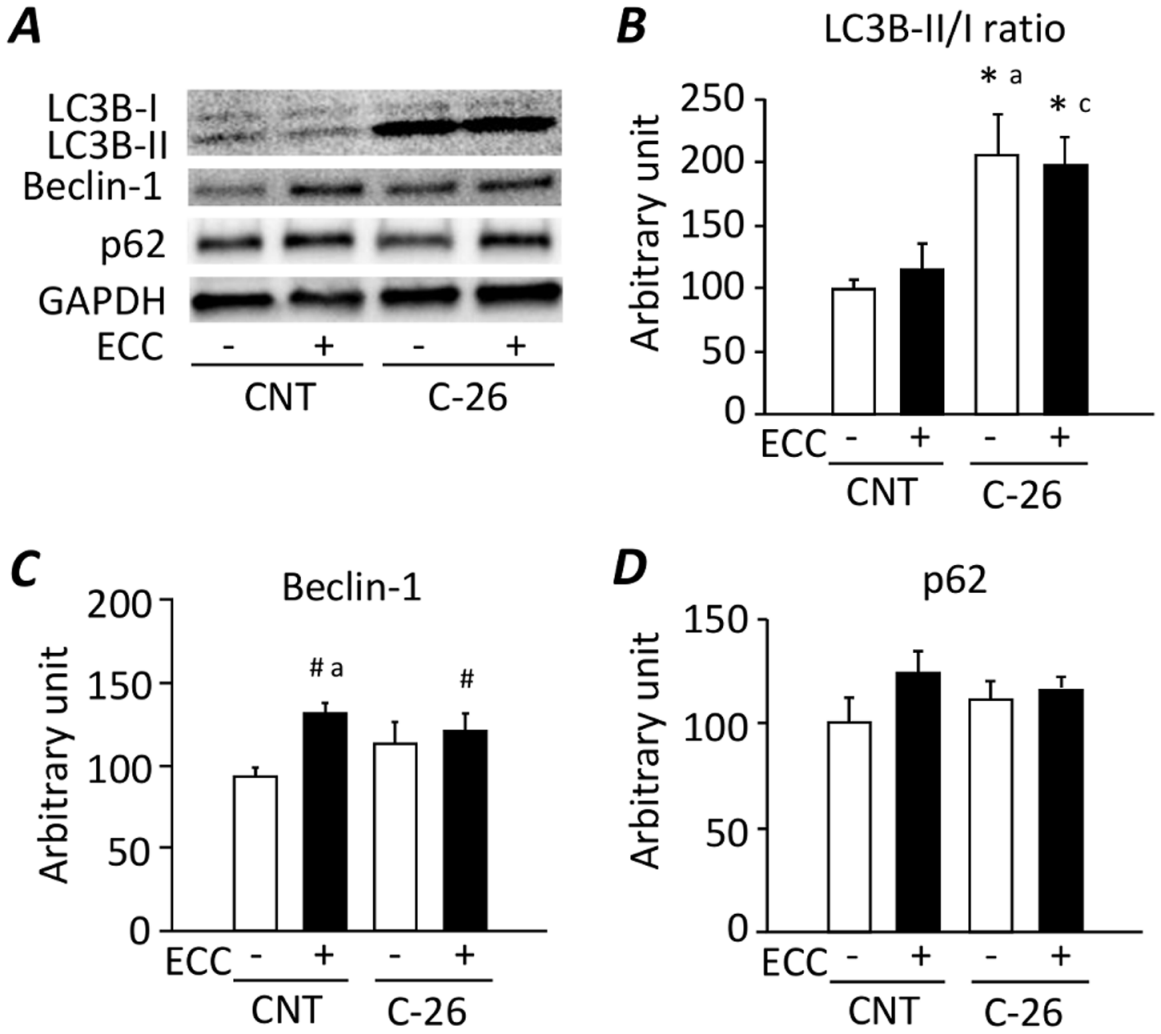
Increase in autophagy-related protein in gastrocnemius muscles from C-26 bearing mice. Representative western blots for microtube-associated protein 1 light chain 3B (LC3B), Beclin-1, and p62 expression in gastrocnemius muscles from control (CNT) and C-26 mice with or without ECC training (***A***). The LC3B-II/LC3B-I ratio (***B***) and the expression levels of Beclin-1 (***C***) and p62 (***D***) normalized to GAPDH content. Data show mean ± SEM for 5 muscles per group. Statistical significance was set at *P* < 0.05: main effect of *C-26 and ^#^ECC; difference versus ^a^CNT-untrained and ^c^CNT-ECC.

The LC3B-II/I ratio in C-26 group was about 2-fold higher than that of CNT group, but it was not reversed by ECC-ES training (Fig 4A & 4B). There was no difference in Beclin-1 expression between CNT and C-26 group (Fig 4A & 4C). Conversely, ECC-ES induced the upregulation of Beclin-1 irrespective of cachexia. The expression levels of p62 did not differ between the groups (Fig 4A & 4D).

### The expression levels of myofibrillar proteins are not altered in gastrocnemius muscles from C-26 bearing mice

**Fig 5:**
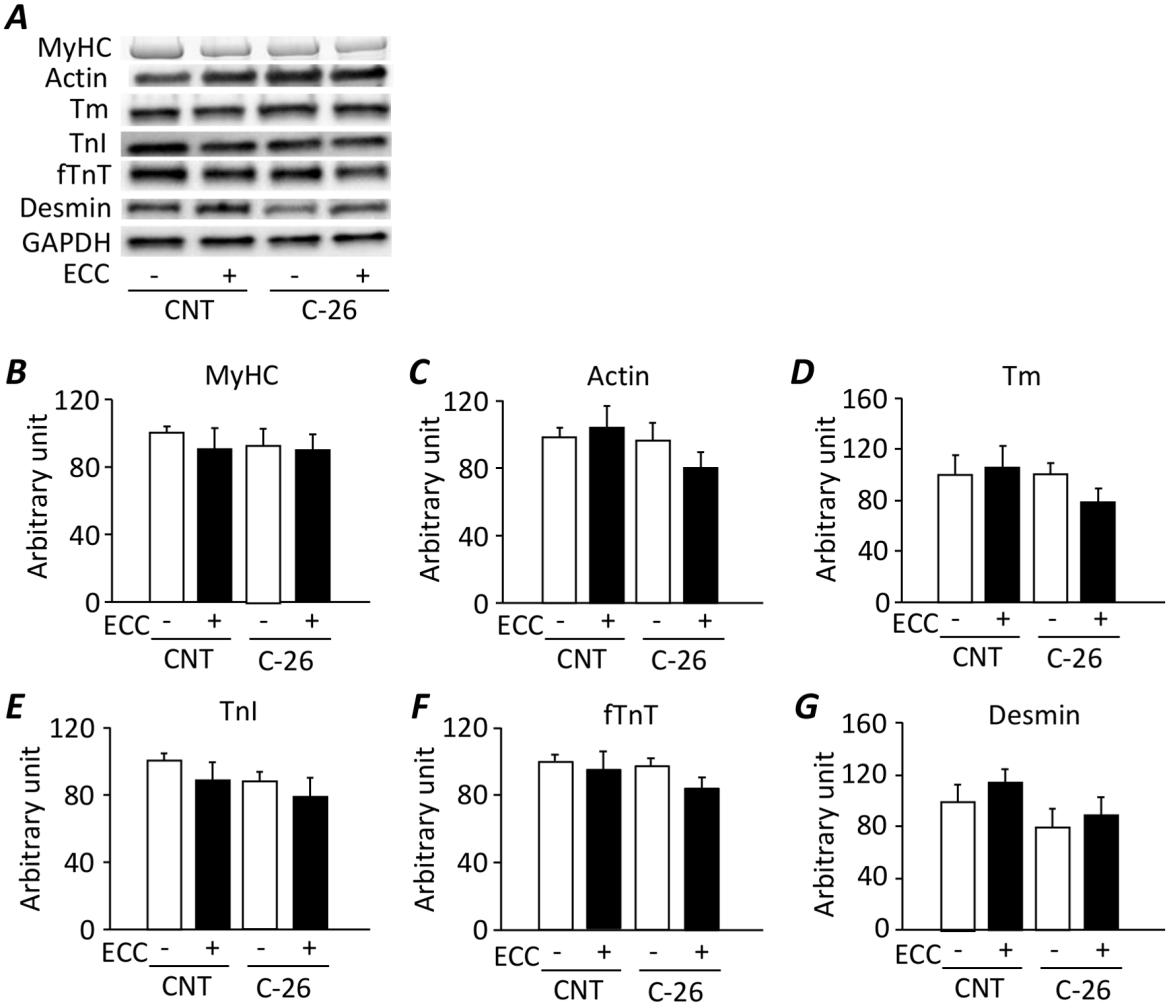
The expression levels of myofibrillar proteins are not altered in gastrocnemius muscles from C-26 bearing mice. Stain free images of myosin heavy chain (MyHC) and representative western blots for actin, tropomyosin (Tm), total troponin I (TnI), fast type troponin T (fTnT), and desmin expression of gastrocnemius muscles in control (CNT) and C-26 mice with or without eccentric (ECC) training (***A***). The expression levels of MyHC (***B***), actin (***C***), Tm (***D***), TnI (***E***), fTnT (***F***), and desmin (***G***) normalized to GAPDH content. Data show mean ± SEM for 5 muscles per group.

The myofibrillar protein concentration in gastrocnemius muscles in each group were as follows: CNT: 80 ± 4 μg/mg; CNT+ECC: 84 ± 1 μg/mg; C-26: 84 ± 2 μg/mg; C-26+ECC: 84 ± 5 μg/mg. There was no difference in myofibrillar protein concentration in gastrocnemius muscles between the groups. Moreover, the expression levels of myofibrillar proteins including myosin heavy chain (MyHC), actin, Tm, TnI, fTnT, and desmin were not altered by tumor-bearing and/or ECC training (Fig 5).

## Discussion

By using a controlled delivery of electrically stimulated ECC, we here show that high-intensity ECC training ameliorates muscle wasting in C-26 mice. The novelty of our study is that this beneficial effects could be mechanistically linked to an activation of protein synthesis and mTORC1 signaling that may be induced in an intensity-dependent manner, despite the presence of the cachexia.

Among the contraction modes, ECC contractions elicit higher torque production than ISO or CONC contractions, and have been regarded as an effective way to increase muscle mass [6]. Importantly, earlier studies on rat skeletal muscles showed that ES-induced augmentation of muscle mass and mTORC1 signaling are determined by loading intensity during muscle contraction regardless of the contraction mode [12, 28]. We and others have reported that ISO- or CONC-ES training failed to prevent muscle atrophy in tumor-bearing mice [13–15]. In contrast, the studies from Carson’s group [13, 14, 29] have shown that repeated ECC-ES can induce fiber growth and the activation of mTORC1 signaling, but not completely restore protein synthesis rate, in cachexic muscles. In these studies, ECC contractions of dorsiflexors were evoked by the electrical stimulation of sciatic nerve, which induces the maximal force production of the plantar flexors that is markedly greater than the dorsiflexors. Therefore, the torque production during ECC contractions was not measured and hence the role of loading intensity in prevention of muscle atrophy under the cachectic condition is incompletely understood [10, 13]. In this regard, the mean peak torque of ECC-ES training in this study was about 5–fold higher than that of ISO-ES training which has not inhibited muscle wasting in C-26 mice (16.4 ± 0.2 mNm vs. 3.4 ± 0.1 mNm) [15]. Thus, our data suggests that the anabolic and regulatory responses are functions of the intensity of mechanical loading even under the cachexia condition.

Importantly, protein synthesis and protein degradation are regulated by pathways that mutually interact [16]. mTORC1 is a crucial component of the anabolic signaling for protein synthesis and is essential for the maintenance of muscle mass and function [30]. It has been shown that the upregulation of MuRF1 is blocked by the activation of protein kinase B, an upstream regulator of mTORC1 activity [31]. Moreover, Shimizu et al. [32] has demonstrated that mTORC1 activation efficiently counteracts the glucocorticoid-induced catabolic processes. Thus, our results of ECC-ES-induced phosphorylation of p70S6K, an indicator of mTORC1 activity, and a reduction in MuRF-1 mRNA expression suggest an inhibitory effects of ECC-ES on ubiquitin-proteasome pathway through mTORC1 activation that were induced by high-intensity mechanical stress in C-26 mice.

ES-induced force production is increased as a result of an increase in the stimulation frequency due to the sigmoidal shape of the force-frequency relationship [33]. Puppa et al. [34] has demonstrated that CONC-ES at LFS induces phosphorylation of p70S6K in control mice, but this induction was blunted by cachexia, suggesting a disruption of anabolic response in cachexic muscles. Conversely, in this study, phosphorylation of p70S6K was induced by ECC-ES at HFS in cachexic muscles that was comparable to control muscles. In addition, this was accompanied by the restoration of protein synthesis rate in C-26 mice. Although several factors could contribute to this inconsistency (e.g., model of cachexia studied and time-point examined), it may be attributable to the difference in the intensity of ES between the studies. Indeed, we have previously shown that ES training at HFS is more effective in inducing muscle hypertrophy and mTORC1 activation than training at LFS with the magnitude of the effects being dependent on the peak torque [12].

In the present study, loss of tumor-free body weight was accompanied by a decrease in gastrocnemius muscle weight in C-26 mice. Glucocorticoid was shown to have antianabolic and catabolic effects [20] and hence result in a net increase in muscle protein degradation especially in fast-twitch fibers. Consistent with these findings [35], our results confirm the activation of ubiquitin-proteasome and autophagy pathway, demonstrated by increased expression levels of muscle specific E3 ubiquitin ligase MuRF-1 mRNA, and lipidated form of LC3B, respectively, in fast-twitch gastrocnemius muscles from C-26 mice. Moreover, these changes were associated with the reduction in protein synthesis rate. Although neither glucocorticoid concentration nor the expression levels of glucocorticoid receptor were not measured in this study, our data suggest that reduction in gastrocnemius muscle weight could result from glucocorticoid-induced negative regulation of the net balance of protein metabolism in C-26 mice.

Preferential loss of myosin has been observed in patients with cancer cachexia [36]. Conversely, myofibrillar protein concentration and the expression levels of MyHC and other myofibrillar proteins were not reduced in C-26 bearing mice. Thus, contrary to the previous study, our results suggest that cachexia-induced muscle atrophy is induced by a nonselective protein degradation. In support, using the C-26 tumor bearing mice, Cosper and Leinwand [37] have shown that MyHC decreases in parallel with other myofibrillar proteins in skeletal muscle. Moreover, although MyHC has been regarded as a specific target of MuRF1 [38], recent findings indicate that MuRF-1 is a general ligase affecting many contractile, soluble, and nuclear proteins [22].

REDD1 is known to be induced by various stressors, including glucocorticoid, and to inhibit anabolic signaling [39]. Correspondingly, we showed that upregulation of REDD1 mRNA was associated with a reduction in protein synthesis rate in muscles from C-26 mice. In the present study, however, ECC-ES increased protein synthesis rate and mTORC1 activity without inhibiting of REDD1 mRNA expression in C-26 mice. These results are inconsistent with the previous study showing that a single bout of ECC-ES represses REDD1 protein expression and elevates protein synthesis rate and mTORC1 signaling in mouse skeletal muscles [40]. The reason for this discrepancy is unclear, but ECC-ES training may augment anabolic signaling independent of REDD1 expression under condition with cancer cachexia.

Our finding of increased LC3B-II/LC3B-I ratio suggests the activation of autophagosome production and/or a delay in autophagosome clearance in muscles from C-26 mice. On the other hand, C-26 tumor bearing did not alter the expression levels of p62, a marker of autophagosome accumulation. Thus, these data suggest an increase in autophagosome production, but not a decrease in autophagosome clearance, in muscles from C-26 mice. In this regard, although beclin-1 is related to autophagy [41], it is debated whether the expression of beclin-1 increases the autophagy induction. In accordance with the present study, it has been shown that starvation-induced autophagy is not accompanied by changes in the expression levels of beclin-1 [42]. Recently, Pigna et al. [35] has demonstrated that voluntary wheel running, an aerobic exercise, counteracts muscle atrophy and restores the levels of autophagic marker in C-26 mice. Conversely, present study showed that preservation of muscle mass induced by the ECC-ES training was not accompanied by an inhibition of increased LC3B-II/LC3B-I ratio in C-26 mice. These findings suggest that ECC-ES training does not inhibit the autophagy in muscles from C-26 mice. Interestingly, activation of autophagy was shown to depend on exercise intensity in human skeletal muscle [43]. Thus, in contrast to an low-intensity aerobic exercise, high-intensity ECC-ES may not reduce autophagosome production in C-26 mice.

## Study limitations

In the present study, ECC-ES training was starting one day after C-26 injection and it effectively ameliorated the muscle wasting in C-26 tumor bearing mouse. It is, however, unclear as to whether ECC-ES can reverse muscle wasting when applied after the induction of wasting. Thus, considering the clinical values of this study, subsequent studies should be performed where ECC-ES is applied after the onset of cancer-induced muscle wasting. Moreover, several conclusions regarding mechanism are based on correlations. In relation to present study, pharmacological or genetic activation of anabolic signaling and/or inhibition of catabolic signaling at the time of C-26 induction would more directly clarify the anti-catabolic and protective effects of ECC-NMES.

Due to practical reasons, we could not repeat the chronic training experiment and hence did not have enough samples to perform the histological analyses including cross-sectional area of the myofibers. The small sample size is the possible limitation of this study.

## Conclusions

We here show that high-intensity ECC-ES training ameliorates skeletal muscle atrophy in C-26 mice, presumably through activation of mTORC1 signaling and the inhibition of ubiquitin-proteasome pathway. These data suggest that even under cachexic conditions, skeletal muscle has the capacity to respond to ES training, with a greater gain at high-loading intensity.

## Supporting information

S1 Fig Original uncropped blots used for Fig 2A. (TIF)

S2 Fig Original uncropped blots used for Fig 4A. (TIF)

S3 Fig Original uncropped blots used for Fig 5A. (TIF)
