# Supplementary figures and images for: High-intensity eccentric training ameliorates muscle wasting in colon 26 tumor-bearing mice
Source: PLoS One. 2018 Jun 12;13(6):e0199050. doi: 10.1371/journal.pone.0199050 (PMC5997314; doi:10.1371/journal.pone.0199050)

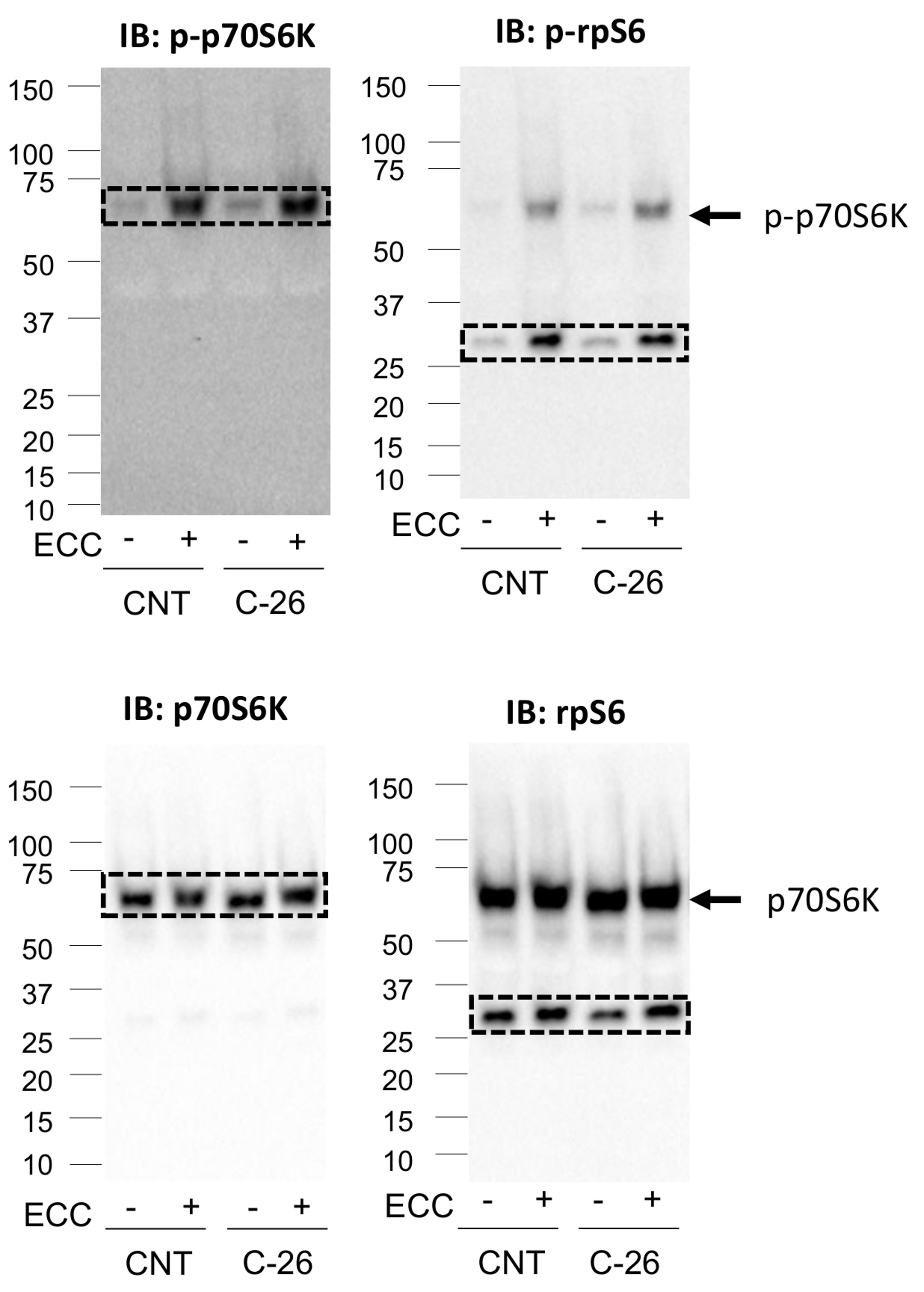

Supplement: S1 Fig — (TIF) [file pone.0199050.s001.tif]

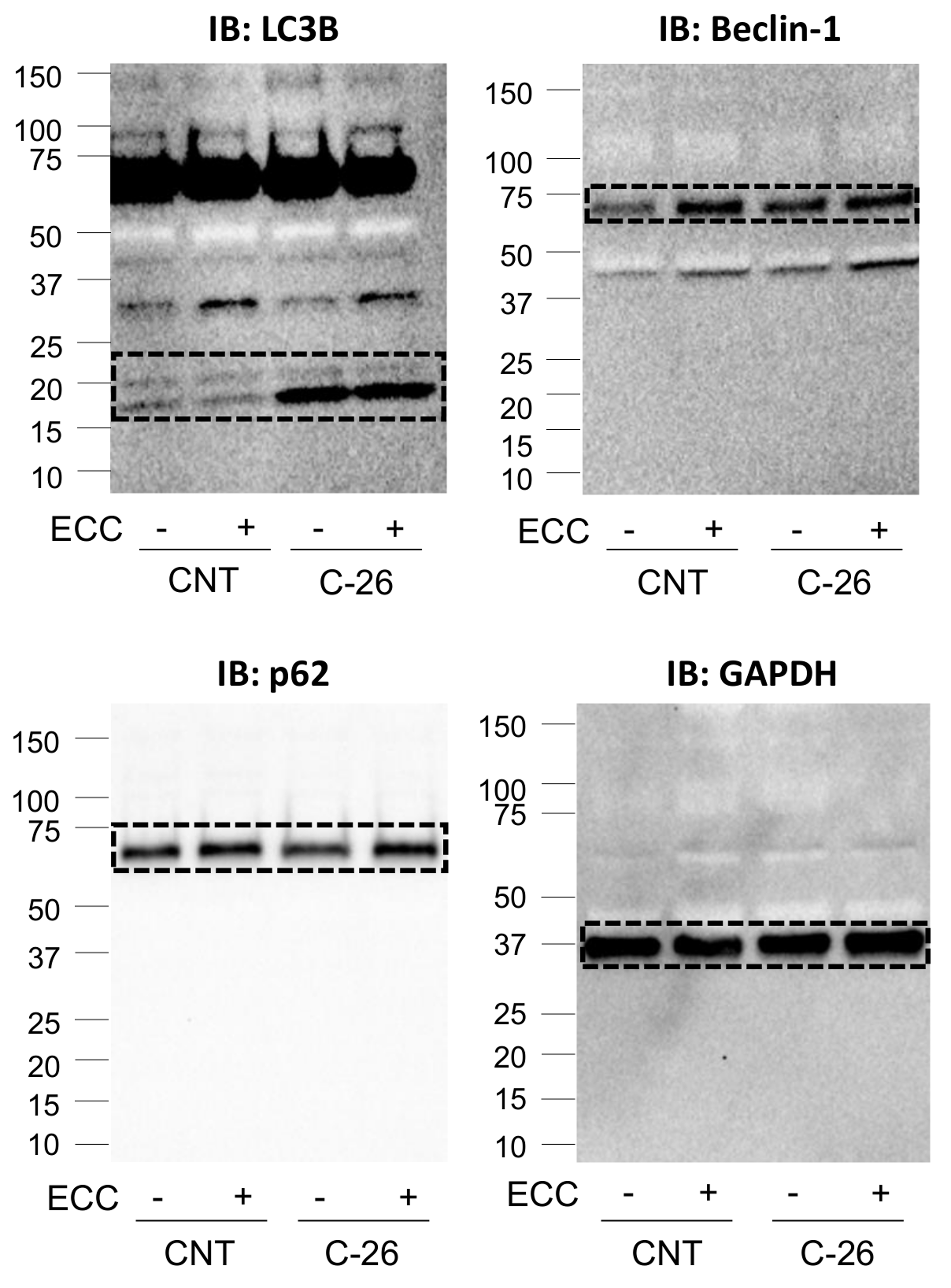

Supplement: S2 Fig — (TIF) [file pone.0199050.s002.tif]

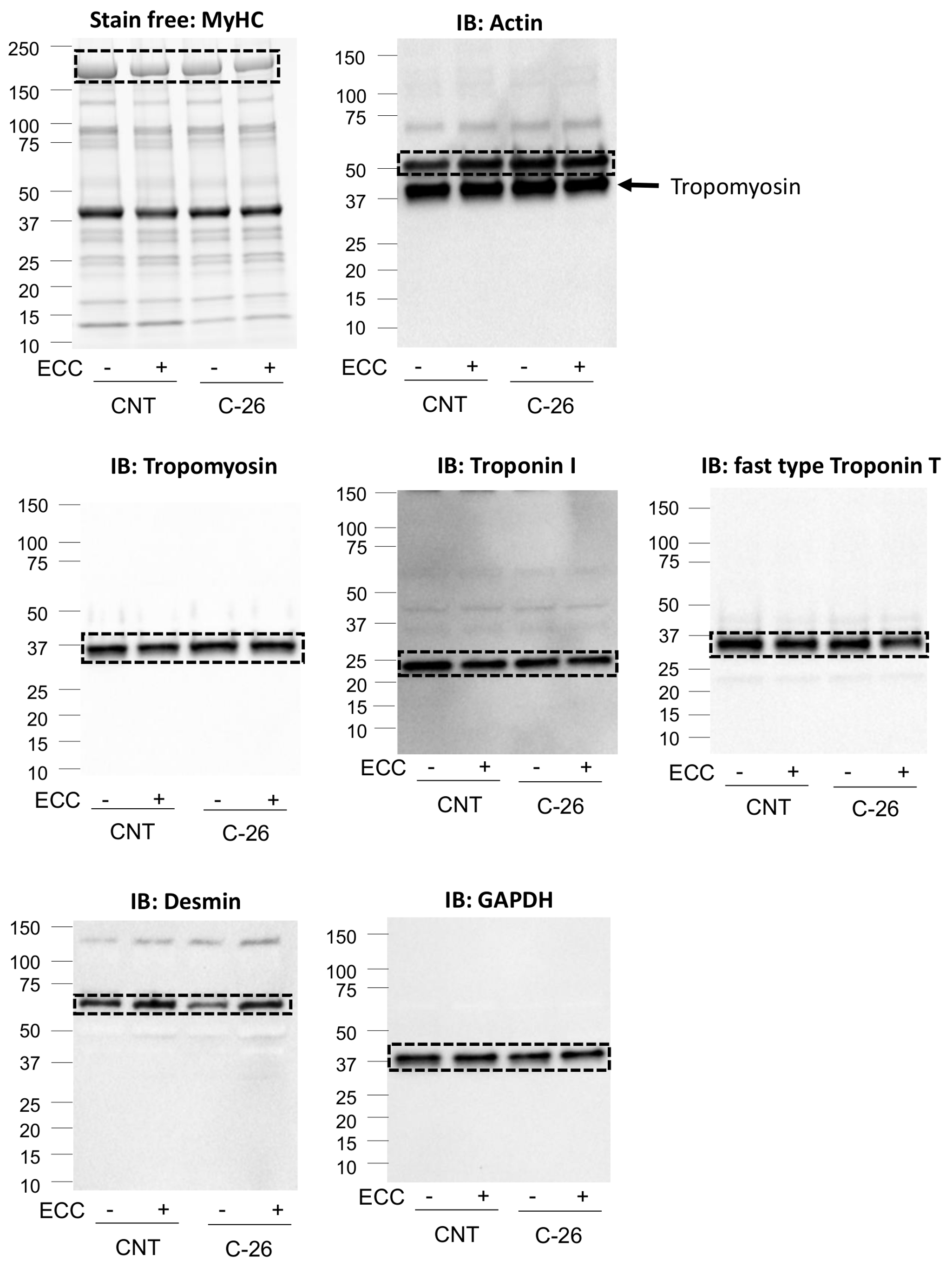

Supplement: S3 Fig — (TIF) [file pone.0199050.s003.tif]
